# Headache in Pregnancy: Preeclampsia and Intracerebral Hemorrhage

**DOI:** 10.7759/cureus.34086

**Published:** 2023-01-23

**Authors:** Zachary Wood, Sarah Adams, Jefferson Jones

**Affiliations:** 1 Family Medicine, Edward Via College of Osteopathic Medicine, Auburn, USA; 2 Family Medicine, Piedmont Columbus Midtown, Columbus, USA; 3 Obstetrics and Gynecology, Piedmont Columbus Midtown, Columbus, USA

**Keywords:** preeclampsia, idiopathic hypertension, hypertension, cesarean section (cs), acute hemorrhagic stroke, cerebral vasospasm, anterior communicating artery aneurysm, spontaneous intracerebral hemorrhage, pre-eclampsia

## Abstract

Preeclampsia is a type of hypertensive disorder of pregnancy that can cause significant maternal and perinatal morbidity and mortality. Hypertension and proteinuria are the keystones of the disease, though systemic end-organ dysfunction may follow. The pathogenesis is multifactorial, with known influences by placental, vascular, renal, and immunological dysfunction. This is a case of preeclampsia complicated by preterm delivery and antepartum intracerebral hemorrhage secondary to aneurysm rupture, presenting as dull headaches and blurry vision, commonly associated with severe features.

## Introduction

This case illustrates a non-traditional presentation and hospital course of a patient with chronic hypertension who developed spontaneous intracranial hemorrhage (ICH) masked by preeclampsia. ICH accounts for 5-12% of all maternal deaths [1]. Unlike overall stroke where approximately 87% of strokes are ischemic and 13% are hemorrhagic [2], up to 66% of maternal strokes are hemorrhagic, including ICH and subarachnoid hemorrhage (SAH) [3]. Those in the postpartum period are at the highest risk of ICH. The risk during the antepartum period parallels a nonpregnant age-related population [4,5]. Most postpartum strokes occur within the first two weeks after delivery, with 50% of readmissions for postpartum strokes occurring within eight days after delivery [6]. Hypertensive disorders of pregnancy, particularly preeclampsia and eclampsia, are the most important risk factors for pregnancy-associated strokes, accounting for 25-57% of maternal strokes [7]. Postpartum headache is a common presentation for patients with peripartum ICH [8]. Usually, an early assessment of postpartum headache is performed by anesthesiologists to rule out headaches correlated with dural puncture. However, an understanding of the risk factors, presentation, and consequences of ICH is crucial. Headaches associated with ICH normally have an acute onset and severity, which contrast the gradual onset and positional pain seen in postdural puncture headaches. Timely identification of a possibly significant underlying etiology of a headache and referral to the appropriate team is imperative, as immediate care may prevent permanent deficits or death.

## Case presentation

**Figure 1:**
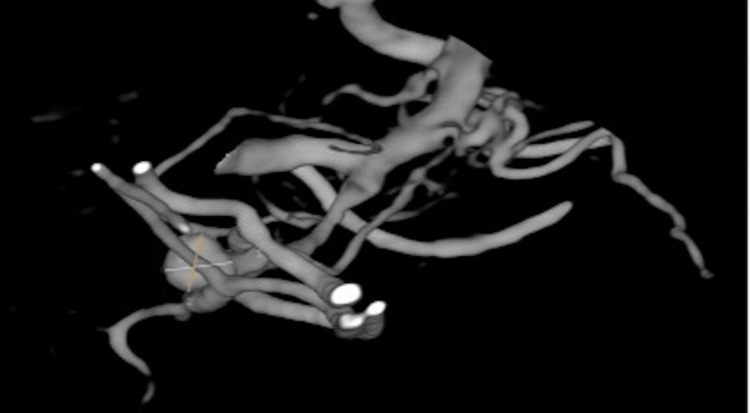
Computed tomography angiography showing an aneurysm measuring 4 x 4 mm

**Figure 2:**
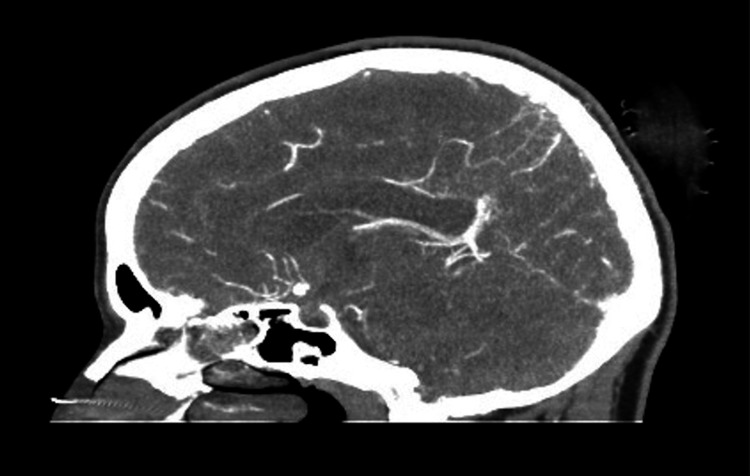
Sagittal computed tomography angiography with contrast

A 29-year-old G1P1 at 24 weeks of gestation with a past medical history of hypertension presented to the emergency department for persistent frontal headaches that she described as dull in nature that began while taking a shower. She complained of intractable back and left leg pain, intermittent blurry vision, and double vision. Obstetric ED workup revealed a 24-hour urine protein of 458 and a ratio of 0.28 with a consistent systolic blood pressure of 150/80, narrowing the diagnosis of preeclampsia with severe features. While admitted to the perinatal unit, she received betamethasone 12 mg, 4 g loading dose of magnesium sulfate followed by 1 g/hour, labetalol 20 mg IV, and hydralazine 10 mg IV. Despite optimal medical therapy and controlled blood pressure, the patient continued to complain of refractory headaches and once again experienced transient double vision. Ophthalmology performed a fundoscopic exam and deemed it inconsistent with papilledema. Neurology assessed the patient; she did not appear to have an intracranial hemorrhage. MRI of the brain revealed no acute findings. It is possible the MRI did not reveal any acute findings, as there was no contrast for an angiogram, which would have revealed an aneurysm and or aneurysmal hemorrhage. The patient was deemed to be preeclamptic by members of the treatment team including obstetrics (OB), maternal-fetal medicine (MFM), neurology, and ophthalmology. On hospital day three, the patient was taken for a low transverse cesarean section (LTCS) using neuraxial anesthesia for persistent headaches and elevated blood pressure despite optimal medical management. She did not experience complications during or immediately after the procedure. She delivered a 1 lb and 6 oz male infant with an Apgar of 6/8, who immediately received care by neonatology. She continued to receive magnesium sulfate after LTCS for 24 hours post-delivery. On postoperative day one, her headaches persisted; however, she did not exhibit any acute changes on the neurologic exam. On postoperative day two, she spiked a temperature max of 102° Fahrenheit, exhibited tachycardia in the low 100s, and had a hypertensive episode that resolved spontaneously after receiving her scheduled medications. Several hours later, she began experiencing nystagmus in her left eye and complained of headaches. CT head and computed tomography angiography (CTA) (Figures 1, 2) were ordered for workup. She also met systemic inflammatory response syndrome (SIRS) criteria and empiric antibiotics were initiated along with evaluation for sepsis. The CT scan revealed a high suspicion of meningitis and the results of the CTA revealed a leaking aneurysm.

The OB team placed a call to a staff neurosurgeon for recommendations, who reviewed scans and recommended immediate transfer to a higher level of care. The patient was transferred within 1.5 hours on magnesium sulfate and nimodipine at the recommendation of the accepting physician and neurosurgery. On arrival, the patient was febrile with a T-max of 103, and she was quickly started on artic sun surface cooling. Neurological examination revealed increasingly abnormal findings; she now exhibited apathy, bilateral lower extremity weakness, and partial abduction paralysis of bilateral eyes. She underwent emergent cerebral angiography and anterior communicating artery (ACA) coiling, complicated by diffuse severe vasospasm during the procedure. This prompted intra-arterial (IA) milrinone in bilateral internal carotid arteries (ICA) and bilateral ACAs. Post-intervention day one, she remained stuporous, on mechanical ventilation, and chemically sedated with propofol for uncontrolled shivering. However, on neurological exam, she was able to follow simple commands intermittently with a significant delay and remained mute. She continued to have excessive urine output despite being started on 3% sodium chloride. Due to the development of intracerebral fluid buildup resulting in hydrocephalus, she required an external ventricular drain placement. Despite previous intervention strategies, she required additional treatment with IA milrinone in bilateral ICAs and left middle cerebral artery (MCA), and subsequent primary angioplasty of the left MCA and left ICA. Repeat CT head showed a stable appearance of the SAH, evolving cerebral edema, and evolving hypodensity in the bilateral orbitofrontal cortex. Her CT pelvis was concerning for endometritis on postoperative day four.

**Figure 3:**
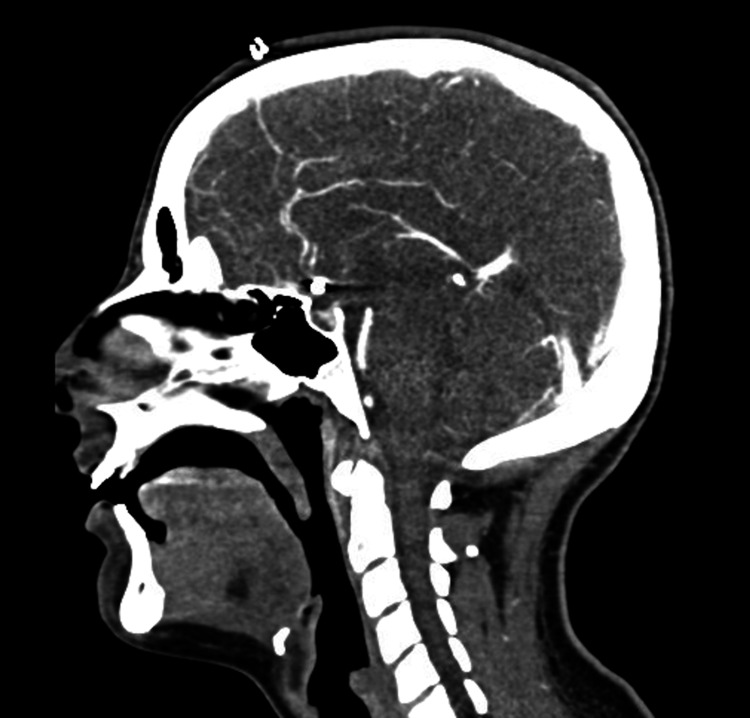
Computed tomography angiography post anterior communicating artery coiling

On postoperative day seven, cerebral angiography findings included diffuse vasospasm in bilateral ICAs and right ACA, prompting escalation of treatment to balloon angioplasty. She tolerated the procedure well, and post-angiography showed resolved radiographic vasospasm. She required additional aneurysmal coiling resulting in the complete obliteration of the aneurysm. After the intervention, she remained abulic and difficult to arouse. MRI brain showed scattered bilateral ACA territory ischemic strokes, and CTA revealed resolved aneurysmal subarachnoid hemorrhage with unchanged ACA coil embolization (Figure 3).

**Figure 4:**
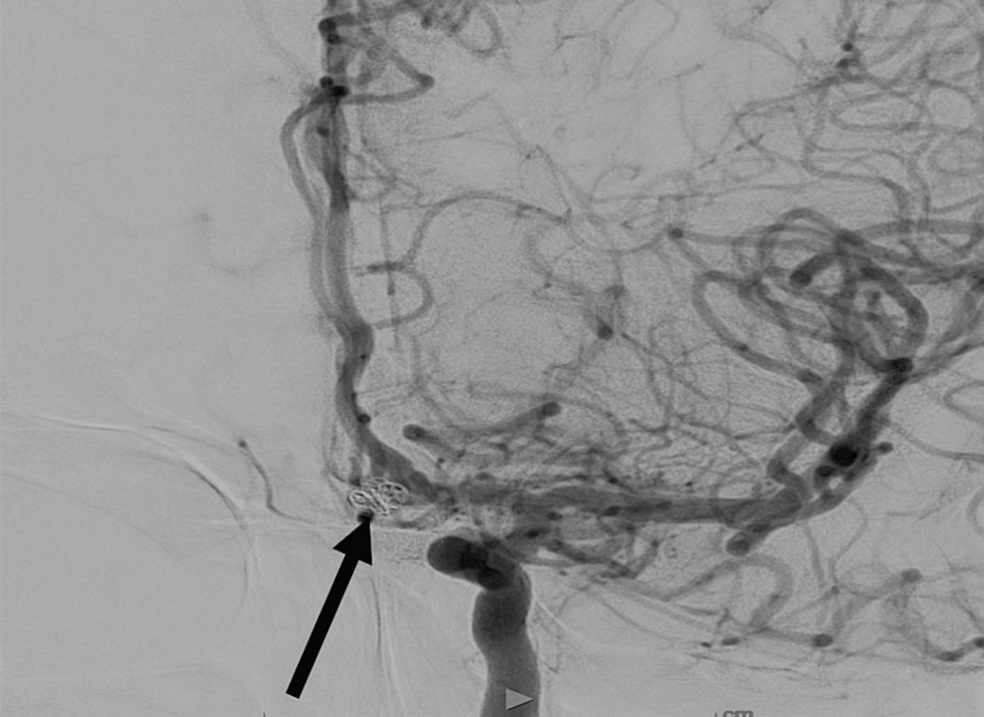
Coronal magnetic resonance angiography showing stable coiling

Luckily, she did not required any further intervention and spent subsequent 18 days in the higher-level care center for medical management before graduating to inpatient acute rehab where she remained on captopril 50 mg three times a day (TID) to restore cerebral blood flow autoregulation through renin-aldosterone system suppression [9]. While in inpatient rehab, she worked with physical therapy and occupational therapy to overcome left-sided lower extremity weakness, numbness, tingling, and proximal left lower extremity pain along with right-sided sciatica over the course of 25 days. She made significant progress during her physiotherapy sessions while inpatient and was transitioned to outpatient day rehab to work on residual cognitive and functional deficits including her ability to perform activities of daily living (ADLs). An outpatient magnetic resonance angiography (MRA) (Figure 4) done three months after her initial admission showed no residual filling within the aneurysm, and she is now receiving outpatient MRAs roughly every three months. She made a near-full recovery, with some muted cognitive deficits and delayed processing of information; however, she learned to perform her ADLs once again including mothering her male infant who spent 100 days in the neonatal intensive care unit.

## Discussion

Stroke contributes to 12% of all maternal deaths [10]. In patients with preeclampsia, when a stroke is present, it accounts for 50% of all deaths related to cerebral complications [11]. Currently, research is still being conducted on women with preeclampsia and other hypertensive disorders of pregnancy to see if treatment should be initiated for the purpose of primary prevention of stroke [12]. The risk of stroke is magnified during the peripartum period due to multiple physiologic features inherent to pregnancy. Increased venous compliance and stasis are seen because of elevated levels of progesterone. Near the end of the third trimester, this reaches a maximum, which parallels the greatest compression on the pelvic veins by the fetus. Higher quantities of procoagulant factors, such as factors VII and X, and prothrombin are also seen due to increased production of estrogen. Additionally, there is an escalation in activated protein C (APC) resistance and a reduction in protein S levels. Plasminogen activator inhibitors are produced by the placenta, which results in a reduction of endogenous tissue plasminogen activator function [13]. Cardiac output increases by 45% with a parallel expansion of intravascular volume by 30-50% throughout pregnancy. It is argued that due to limited evidence, these changes discussed above could have an influence on the enlargement of cerebral aneurysms and brain arteriovenous malformations [14,15]. Thus, the vulnerability during the peripartum and postpartum period to hemorrhagic and ischemic stroke may be due to these physiological changes in the cerebral vasculature. Cerebral arterial remodeling and capillary proliferation in end-stage pregnancy have been shown in animal studies [16]. Increased blood-brain barrier permeability, neuroinflammation, and cerebral autoregulatory dysfunction were seen in pre-eclamptic rat studies, perhaps leading to the increased predisposition to both hemorrhagic and ischemic stroke [17,18].

## Conclusions

Maternal morbidity and mortality are both climbing in the US, and maternal stroke is an increasingly critical cause. Our comprehension of the aspects and features that may trigger maternal stroke is not clear and contains many holes. Although several risk factors have been recognized for maternal stroke, there are no calculations or formulas to aid in identifying which patients may be in the greatest danger of postpartum stroke. This can allow us to categorize which women necessitate closer monitoring. Additionally, estimations of the risk of maternal stroke, particularly recurrent stroke, have not been sufficiently quantified. The multifaceted pathophysiology of pre-eclampsia and other hypertensive disorders of pregnancy, and its consequences on cerebral vasculature, demand further research and investigation. Reducing maternal morbidity and mortality requires a dire focus on the risk factors, identification, and interventions of maternal stroke.
